# Supplementary material for: Murine Leukemia Virus GlycoGag Antagonizes SERINC5 via ER-phagy Receptor RETREG1
Source: bioRxiv. 2025 Mar 6:2025.03.06.641798. Preprint. [Version 1] doi: 10.1101/2025.03.06.641798 (PMC11908239; doi:10.1101/2025.03.06.641798)
Supplement: Supplement 1 [file NIHPP2025.03.06.641798v1-supplement-1.pdf]

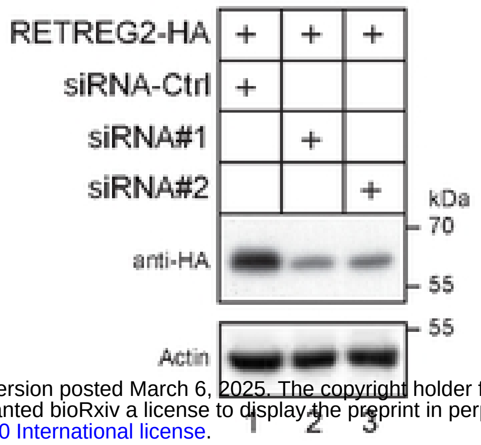

bioRxiv preprint doi: https://doi.org/10.1101/2025.03.06.641798; this version posted March 6, 2025. The copyright holder for this preprint (which was not certified by peer review) is the author/funder, who has granted bioRxiv a license to display the preprint in perpetuity. It is made available under aCC-BY 4.0 International license.

Figure S1. RETREG2 was expressed with its specific siRNAs (#1, #2) or a control (Ctrl) in HEK293T cells and its expression was detected by WB.

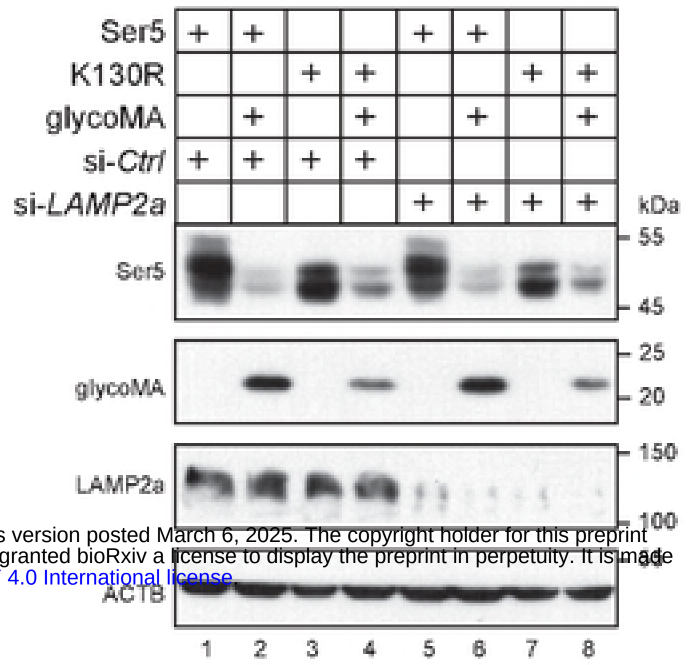

bioRxiv preprint doi: https://doi.org/10.1101/2025.03.06.641798; this version posted March 6, 2025. The copyright holder for this preprint (which was not certified by peer review) is the author/funder, who has granted bioRxiv a license to display the preprint in perpetuity. It is made available under aCC-BY 4.0 International license.

Figure S2. LAMP2a is not required for glycoMA downregualtion of Ser5. Ser5 and K130R were expressed with glycoMA in HEK293T cells in the presence of *LAMP2a*-specific siRNA or its control (Ctrl). Protein expression was detected by WB.
